# The transcriptome of retinal Müller glial cells

**DOI:** 10.1002/cne.21730

**Published:** 2008-07-10

**Authors:** Karin Roesch, Ashutosh P Jadhav, Jeffrey M Trimarchi, Michael B Stadler, Botond Roska, Ben B Sun, Constance L Cepko

**Affiliations:** 1 Department of Genetics, Harvard Medical SchoolBoston, Massachusetts 02115; 2 Howard Hughes Medical InstituteBoston, Massachusetts 02115; 3 Friedrich Miescher Institute for Biomedical Research4058 Basel, Switzerland; 4 Canaccord AdamsBoston, Massachusetts 02110

**Keywords:** murine retina, Müller glia, microarray, molecular markers, BAC transgenic mouse, transcriptome

## Abstract

Müller glial cells are the major type of glia in the mammalian retina. To identify the molecular machinery that defines Müller glial cell identity and function, single cell gene expression profiling was performed on Affymetrix microarrays. Identification of a cluster of genes expressed at high levels suggests a Müller glia core transcriptome, which likely underlies many of the functions of Müller glia. Expression of components of the cell cycle machinery and the Notch pathway, as well as of growth factors, chemokines, and lipoproteins might allow communication between Müller glial cells and the neurons that they support, including modulation of neuronal activity. This approach revealed a set of transcripts that were not previously characterized in (Müller) glia; validation of the expression of some of these genes was performed by in situ hybridization. Genes expressed exclusively by Müller glia were identified as novel markers. In addition, a novel BAC transgenic mouse that expresses *Cre* in Müller glia cells was generated. The molecular fingerprint of Müller glia provides a foundation for further studies of Müller glia development and function in normal and diseased states.

Glial cells are found throughout the central nervous system. In the mammalian retina, three types of glia have been identified: Müller glia, astrocytes, and microglia. Astrocytes originate in the more medial aspects of the brain, and enter into the retina through the developing optic nerve (Stone and Dreher, 1987; Watanabe and Raff, 1988). Microglial cells are thought to be of mesodermal origin (Chan-ling, 1994). The only glial cells derived from the retinal neuroepithelium are the Müller glia. They arise late in development from multipotent progenitor cells (Sidman, 1961; Young, 1985; Turner and Cepko, 1987). In the mature retina, they are normally not mitotic but can re-enter the cell cycle under certain circumstances (Sahel et al., 1990; Dyer and Cepko, 2000).

Müller glial cells are radial and form the outer and inner limiting membranes. They surround neuronal cell bodies and processes within the retina and perform a wide range of functions, including structural stabilization, regulation of ion homeostasis, neurotransmitter recycling, and neuronal survival (Newman and Reichenbach, 1996; Bringmann et al., 2000; Mata et al., 2002). Recently it was shown that their specific cylindrical shape, their regular and parallel orientation within the retina, and the high refractive index of their stalk allow them to capture and transfer light efficiently through the retina (Franze et al., 2007). Müller glia can undergo reactive gliosis, when they divide and upregulate glial fibrillary acidic protein (GFAP) in response to neuronal damage (Ridet et al., 1997; Pekny and Pekna, 2004). Gliotic Müller glial cells also show altered expression of various enzymes, ion channels, and receptors. The significance of this response is poorly understood. The role of Müller glial cells in disease has become even more intriguing, because an intimate molecular and functional relationship between Müller glia and multipotent progenitors has become appreciated in various systems, leading to the suggestion that Müller glia might serve as a source of retinal neurons following injury (Fisher and Reh, 2001,^2003^; Ooto et al., 2004; Blackshaw et al., 2004, Osakada et al., 2007).

Although extensive studies of the physiological and morphological properties of Müller glia have been performed (Reichenbach and Robinson, 1995; Newman and Reichenbach, 1996), much less is known of their molecular biology. In the retina, gene expression in the abundant rod photoreceptors is most well characterized (Blackshaw et al., 2001), and advances have been made in understanding the molecular nature of specific neuronal cell types such as amacrine and ganglion cells (Ivanov et al., 2006; Trimarchi et al., 2007). Considerably less is understood about the molecular complexity of Müller glia. In this study, we have employed microarray analysis of single cells to obtain a detailed profile of the Müller glial transcriptome. Expression of Müller glia enriched or specific transcripts was tested by in situ hybridization on retinal sections and on dissociated retinal cells. Many novel Müller glial genes have been found. Clusters of genes with characteristics related to Müller glial cell function, such as K+ and water homeostasis, neuronal signaling, and recycling of photopigments, were readily identified.

## MATERIALS AND METHODS

### Isolation of single Müller glial cells and cDNA amplification

Single-cell cDNA generation was achieved as described (Brady and Iscove, 1993; Dulac and Axel, 1995; Tietjen et al., 2003; Trimarchi et al., 2007). In short, retinae from C57/BL6 adult mice were dissected in phosphate-buffered saline (PBS; pH 7.4) and then dissociated with papain (Worthington Biochemical, Freehold, NJ) for 15–20 minutes at 37°C and gentle manual trituration. Cells were pelleted and resuspended in PBS containing 0.1% bovine serum albumin (BSA). Dissociated cells were visualized under an inverted microscope, and Müller glial cells were identified based on their distinct morphology. They were transferred with a drawn glass needle into wash buffer (PBS/0.1% BSA) and then seeded into polymerase chain reaction (PCR) tubes containing cold lysis buffer. cDNA was generated from single cells by reverse transcription (Superscript II [Invitrogen, La Jolla, CA]). After poly(A) tailing of the synthesized first-strand cDNA product, PCR amplification of the samples was performed by using LA Taq polymerase (Takara Mirus Bio, Shiga, Japan; Trimarchi et al., 2007). The cDNA smears were analyzed on agarose gels.

### Affymetrix array hybridization

Ten micrograms of each single-cell cDNA were digested for 13 minutes at 37°C with 1 U of RNase free DNase I (Roche, Indianapolis, IN) in One-Phor All buffer (100 mM Tris acetate, 100 mM Mg acetate, 500 mM K acetate). Dnase I was heat-inactivated, and the samples were labeled with 25 μM biotin N6 ddATP (Enzo Biosciences, Syosset, NY) by using 45 U of TdT (Roche; Tietjen et al., 2003).

Mouse genome 430 2.0 Genechip® oligonucleotide arrays were prehybridized in a Genechip® Hybridization Oven 320 (Affymetrix, Santa Clara, CA) in 1X MES buffer (100 mM MES, 1 M NaCl, 20 mM EDTA, 0.01% Triton X-100) with 0.5 mg/ml acetylated BSA and 0.1 mg/ml herring sperm DNA. The single cell cDNAs were heated to 99°C for 5 minutes, cooled to 45°C, added to the microarrays, and incubated in the hybridization oven at 45°C for 16 hours. The slides were then scanned on a Genechip Affymetrix Scanner 7G. Signals from microarrays were globally scaled with a target intensity set to 500 by using Affymetrix Microarray Suite Software (MAS 5.0). Signal levels for all Affymetrix probe sets on the mouse 430 2.0 microarray are listed in Supplemental Table T4 for the five Müller glial cells profiled in this study as well as the 23 single cells (21 immature cells, 2 adult rod photoreceptors) used for comparison.

### Data analysis: hierarchical clustering and Fisher's exact test

Gene Cluster software (Eisen et al., 1998) was used to determine gene association by hierarchical clustering. The results were visualized with Treeview software (Eisen et al., 1998). The data were filtered such that any probe set that failed to reach a signal of 1,000 in any single cell was eliminated; 1,000 was found to be a distinct value, where the threshold of a present and absent call on the array was very clear. The data for the remaining probe sets were log transformed and normalized according to the software instructions.

Additionally, for each probe set that was detected with a signal of >1,000 in at least one microarray, the expression values were scaled by dividing each value by the maximum value for that probe set. The resulting scaled values were then binned into five equally sized bins as described (Trimarchi et al., 2007). All probe set pairs were analyzed for association by using the following procedure. First, a contingency table with five rows and five columns was obtained that recorded the joint distribution over bins for a given probe set pair. A *P* value for significant association was then calculated from this table by using Fisher's exact test. For multiple Affymetrix identifiers that correspond to a single gene, the *P* values were generated as follows. First, for each gene, all Affymetrix IDs that map to this gene were identified. Then, for each microarray, the maximal expression value for these Affymetrix IDs was identified. This maximal value was then used as the expression value for that gene in that array. This collapsing method is referred to as “each-max-value”. A new ID was then generated by a comma-separated list of all Affymetrix IDs that were collapsed for a given gene. For each array and gene, the maximal value among the Affymetrix IDs was always used. The resulting expression profile of a gene was thus a combination of values measured by any of the collapsed Affymetrix probe sets and therefore does not always correspond to a single Affymetrix probe set profile.

### In situ hybridization, Xgal histochemistry, and immunohistochemistry

RNA tissue hybridization was performed following the detailed protocol described (Trimarchi et al., 2007), which is based on previous techniques developed by Murtaugh et al. (1999). Retinae were dissected from young adult (>P21) C57/BL6 or CD1 mice. Tissues were fixed in 4% paraformaldehyde (PFA) in PBS (pH 7.4), dehydrated, and embedded in paraffin, or cryoprotected in 30% sucrose in PBS and embedded in Tissue-Tek OCT compound. In situ hybridization (ISH) was performed on 20-μm retinal sections. The protocol used for dissociated cell in situ hybridization (DISH) was performed as in Blackshaw et al. (2004) and Trimarchi et al. (2007), in combination with immunohistochemistry. For antibody incubation and detection, slides were incubated in blocking buffer (PBS, 0.1% Triton-X100, and 5% goat serum) for 30 minutes at room temperature in a humidified chamber. Primary antibody (anti-Glul, mouse monoclonal antibody against glutamine synthetase; Chemicon/Millipore, Temecula, CA, MAB302; as immunogen glutamine synthetase purified from sheep brain) was added 1:500 diluted in PBS and incubated at room temperature for 1.5 hours. This antibody recognizes a single 45-kDa protein in adult retinal tissue by Western blot analysis (Chang et al., 2007), and staining of adult murine retinal sections revealed a characteristic glial expression pattern (Rhee et al., 2007).

Slides were then washed three times with PBT (PBS and 0.1% Tween-20), and then cy2-labeled secondary antibody (donkey anti-mouse IgG, cyanine cy2, Jackson ImmunoResearch, West Grove, PA, 715-225-150) in PBT (1:250) was added. The slides were incubated at room temperature for 1 hour and afterwards washed again three times with PBT. At the end, slides were DAPI-stained and mounted in gel/mount (Biomeda, Foster City, CA). For immunohistochemistry on 20-μm OCT retinal sections, slides were blocked in blocking buffer (PBS, 0.1% Triton-X100, and 10% goat serum) for 45 minutes at room temperature in a humidified chamber. Primary antibody, polyclonal anti-Pax6 (purified rabbit antibody; Covance, PRB-278P; generated against the peptide QVPGSEPDMSQYWPRLQ of the mouse Pax6 protein and purified on a peptide affinity column; Marquardt et al., 2001), and monoclonal anti-Glul (Chemicon/Millipore, MAB302) were added at 1:150 and 1:500, respectively, diluted in blocking buffer, and incubated overnight at 4°C. The anti-Pax6 antibody reacts strongly with a single band of approximately 50 kDa on Western blots of retinal tissue extracts (Davis and Reed, 1996).

Slides were then washed three times with PBT (PBS and 0.1% Triton-X100), and then cy2-labeled anti-mouse secondary antibody (Jackson ImmunoResearch, 715-225-150) and cy3-labeled anti-rabbit secondary antibody (donkey anti-rabbit IgG, cyanine cy3, Jackson ImmunoResearch, 711-165-152) in blocking buffer (1:250) were added. The slides were incubated at room temperature for 3 hours and afterwards washed again three times with PBT. Slides were DAPI-stained and mounted in gel/mount. For Xgal staining, retinal tissue was harvested and fixed with a 1% PFA/0.5% glutaraldehyde mix (Kwan et al., 2001). Retinae were stained as whole mounts (Turner et al., 1990) and sectioned afterwards.

### Photography

Photography of immunohistochemical staining patterns on sections was done on an upright confocal microscope (Leica TCS SP2). Pictures of ISH and DISH experiments were acquired on either a Zeiss Axiophot microscope or a Nikon eclipse E1000 microscope, both equipped with a Nikon DXM 1200F digital camera. Brightness and contrast of the section ISH and DISH images were adjusted by using Adobe Photoshop 7.0 (San Jose, CA). Digital magnification was also done by using Adobe Photoshop 7.0.

### Generation of Pdgfra BAC transgenic mice

The *Cre* recombinase gene, followed by the SV40 late polyA site and an FRT-flanked kanamycin cassette, was cloned into a pBSKS vector (Cre-pA-kan). Cre-pA-kan was amplified by PCR primers containing the following 50-bp homology arms and *Not*I sites (Lee et al., 2001; Liu et al., 2003): CGCCCCGCGGCCGCGGCTAATGCTGTTTATGTTTGGCTTTTGTCATTTGCAGGTCTCAGGAGCTATGCCCAAGAAGAAGAGGAAGGTGTCCAA and ATAATTGCGGCCGCATACCTGTGAGGAGACAGCTGAGGACCAGAAAGACCTGGTGGGAGGTCCCTCCCGGCGGATTTGTCCTACTCAGGAGAGCG. The PCR product was digested by *Not*I and cloned into the *Not*I sites of pBSKS (pdg-Cre-pA-kan-pdg). The pdg-Cre-pA-kan-pdg was digested by *Not*I and recombined into the RP23-116F7 BAC clone; the loxP sites of the recombined BAC were removed, and the BAC was linearized with *Asc*I before pronuclear injection (Lee et al., 2001; Liu et al., 2003). The BAC was injected into C57/BL6 blastocysts, and transgenic animals were afterwards crossed to C57/BL6 animals.

### Animals

CD1 and C57/BL6 mice were purchased from Charles River Laboratories (Wilmington, MA). The JAX® transgenic strain B6.129P2-*Gpr37*^*tm1Dgen*^/J was purchased from the Jackson Laboratory (Bar Harbor, ME). Mice were genotyped for the targeted allele by using DNA extracted from tail preparations, and PCR amplification with the following primer pairs was performed. For the targeted allele, the gene-specific 5′ primer, GGCCAAGAGAGAATTGGAGATGCTC, and the 3′ primer in neo, GGTGGATTAGAGCTTAAATGCCTGCTCT, were used. For the endogenous allele, the gene-specific 5′ primer, GGCCAAGAGAGAATTGGAGATGCTC, and the gene-specific 3′ primer, AACGGGTCTGCAGATGACTGGGTTC, were used.

Mice carrying the *Pdgfra-CRE BAC* transgene or the *RC::PFwe* allele (gift of A. Farago and S. Dymecki; Farago et al., 2006) were crossed to generate mice hemizygous for *PDGFRa-CRE* and hemizygous for the *RC::PFwe* allele.

## RESULTS

### Gene expression profile of single Müller glial cells

**Fig. 1:**
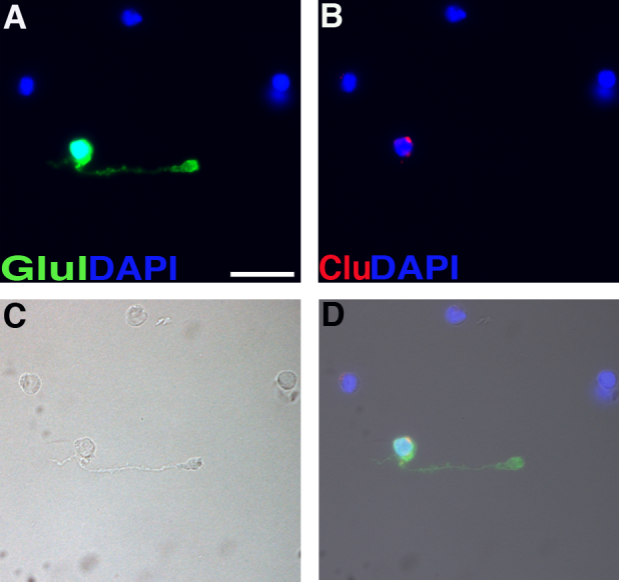
Prospective identification of single Müller glial cells by morphology. Micrograph of cells dissociated from an adult mouse retina. **A**: Immunostaining for glutamine synthetase (Glul). **B**: Dissociated cell in situ hybridization for clusterin. **C**: Brightfield image. **D**: Merged image. Scale bar = 10 μm in A (applies to A–D).

**Fig. 2:**
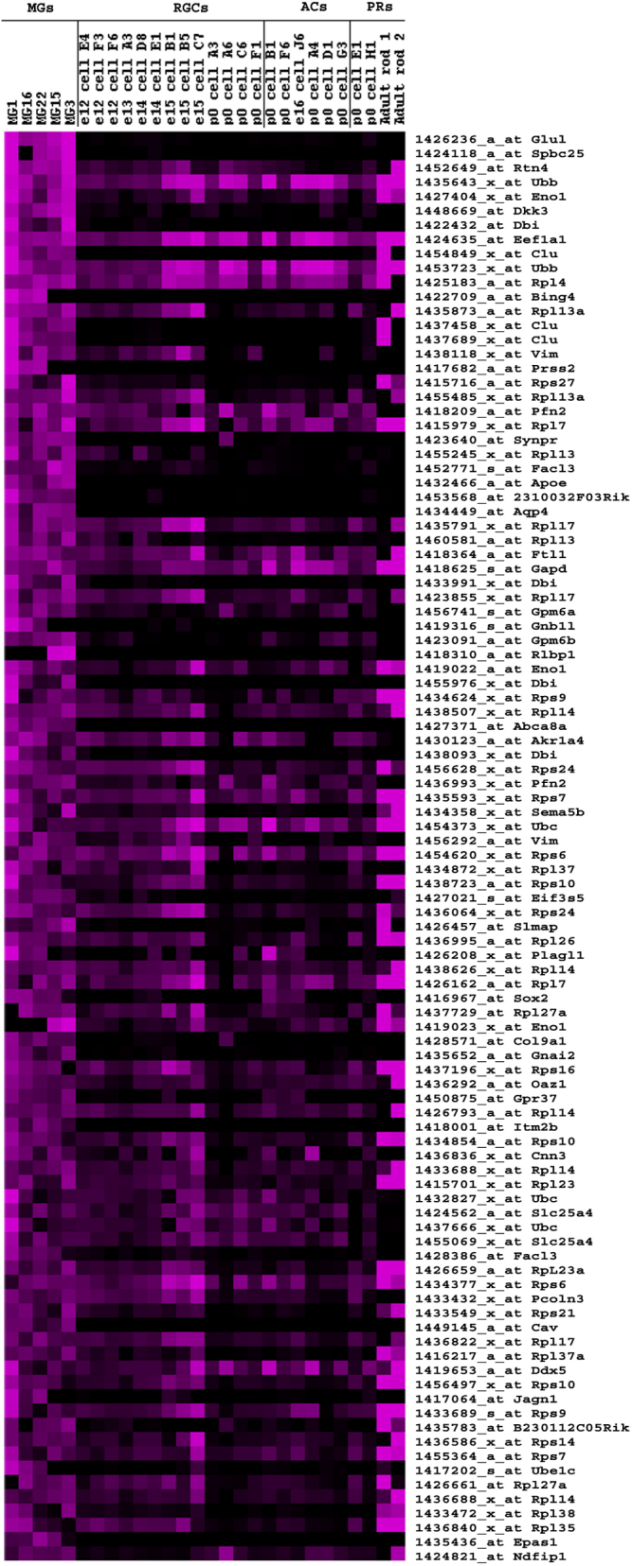
Comparison of highly expressed transcripts in Müller glia with other retinal cell types. The 100 most highly expressed transcripts, averaged over the five single Müller glial cells (MGs) and sorted in decreasing order, are illustrated in a heat map and compared with their expression in other cell types. Values above 10,000 are represented in dark purple. Rows correspond to different genes (with abbreviated name and Affymetrix ID), and columns represent the single cell probes. Shown are 19 immature amacrine (ACs) and ganglion cells (RGCs), 2 immature rod photoreceptors (PRs) from postnatal day 0 (P0), and 2 mature rod photoreceptors (PRs) (Trimarchi et al., 2007).

Adult murine retinae were dissociated to single cells by using papain digestion. Within less than 2 hours of dissection, individual Müller glial cells were identified by their distinctive morphology (Fig. 1) and picked from a dish of dissociated cells with a micropipette. They were then washed, lysed, and subjected to reverse transcription. After a terminal transferase reaction to add A's to the 3′ end of the cDNA, 35 rounds of PCR were carried out by using modified oligo dT primers. cDNA preparations from five individual Müller glial cells were hybridized to Affymetrix Genechip® Mouse genome 430 2.0 arrays. These arrays provide almost complete coverage of the mouse genome with 45,000 probe sets. The identity of cells as Müller glial cells was confirmed by expression of known marker genes for Müller glia. Glutamine synthetase (Glul), clusterin (Clu), dickkopf homolog 3 (Dkk3) (Blackshaw et al., 2004), and S100 calcium binding protein A16 (Seigel et al., 1996) were successfully detected (Fig. 2 and Supplemental Fig. S1). All of the cells share expression of these key marker transcripts.

The values for the Affymetrix signals from the five Müller glial cells were averaged (Supplemental Table T2). There were 7,377 Affymetrix IDs with averages > 1,000, of which 871 had values > 10,000. Many known Müller glial specific genes, such as Glul, aquaporin 4 (Aqp4), and Clu/ApoJ were some of the most highly expressed genes (Fig. 2), perhaps facilitating their previous discovery as Müller glial markers (Jenne and Tschopp, 1992). Additionally, this comparison of highly expressed genes with the data in the literature (Nagelhus et al., 1998; Blackshaw et al., 2004) validates the general performance of the assay.

Biological processes and molecular functions were systematically assigned to genes with average expression values > 10,000 by using IPA (Ingenuity Systems, Redwood City, CA). Genes with the highest significance can be categorized into protein synthesis, cell-to-cell signaling, cellular assembly and organization, cellular function and maintenance, carbohydrate metabolism, cell morphology, and lipid metabolism (Supplemental Table T6).

### Comparison of expression of Müller glia enriched transcripts to expression in other retinal cells

The Affymetrix signal values from the Müller glial cells were compared with those of several different types of single retinal single cells in order to gain an appreciation of how Müller glial cells differ from neurons and also to identify markers for Müller glial cells. This comparison included 2 immature rod photoreceptors, 2 adult rod photoreceptors, and 19 immature amacrine and ganglion cells (Trimarchi et al., 2007). The data were sorted according to decreasing average values from the five Müller glia samples (Supplemental Table 1) and are represented in Figure 2 as a heat map. The 100 transcripts expressed with the highest averaged values show an almost uniform general expression pattern across all Müller glial samples. Many genes that had high expression values in Müller glia were also highly expressed in rod photoreceptors and immature amacrine and ganglion cells. Some of these genes are involved in generic cellular functions, e.g., ribosomal subunits, eukaryotic translation elongation factor 1 α 1 (Eef1a1), ubiquitins (Ubb and Ubc), and glyceraldehyde-3-phosphate dehydrogenase (Gapd).

This initial comparison revealed a dozen genes that appear to be preferentially expressed by Müller glia, including the tRNA ligase BING4 and the transcription factor Sox2. They also include the lipid transporter ApoE, the major apolipoprotein in the central nervous system (Pitas et al., 1987). Interestingly, ApoE has also been implicated in cell proliferation (Mahley, 1988) and in modulation of the innate and acquired immune response (Laskowitz et al., 2000). Another gene involved in lipid metabolism that was found enriched in Müller glia is the diazepam binding inhibitor (Dbi), which is also known to be expressed in glial cells of both the central and peripheral nervous systems (Yanase et al., 2002). Other Müller glia enriched genes are the water channel, Aqp4, and Abca8a, a transporter with ATPase activity, the isoprenoid binding protein Rlbp1, and the receptors Gnai2 and Gpr37. Gnai2 (also called Gi2) mediates signaling from the endothelin-B receptor to maintain mitogenic activity of neural progenitors in the brain (Sinohara et al., 2004).

Expression of the Gpr37 and Dkk3 transcripts in Müller glia was shown in a previous study using serial analysis of gene expression (SAGE; Blackshaw et al., 2004). Dkk3 is a gene involved in cell differentiation and negative regulation of Wnt signaling. Further, caveolin1 (Cav), which is important in nitric oxide metabolism and vesicle-mediated transport, Spbc25, a component of the Ndc80 kinetochore complex (McCleland et al., 2004), and genes with unknown biological function such as Itm2b, Jagn1, and 2310032F03Rik can be classified as enriched in Müller glia. The data suggest that these genes are specific for Müller glia in the adult murine retina. However, some genes are also expressed in retinal progenitor cells, as has been shown for Sox2 and Dkk3 (Blackshaw et al., 2004), and for Spbc25 (data not shown; Trimarchi and Cepko, in preparation). Müller glia enriched transcripts for synaptoporin (Synpr) and type IX procollagen alpha1 (Col9a1) might also be Müller glia specific, although both of these are also detectable in one immature ganglion cell (P0 A6). Some of the potentially Müller glia specific transcripts, such as BING4, Prss2, Ube1c, and Gnb1l could only be detected in three of five Müller glial cells. This might be an indication of heterogeneity within Müller glial cells, or it might be due to a technical issue.

The Müller glia marker clu, expected to be specific to Müller glia in this comparison, is also detected in one of the rod photoreceptors. Because the retinal pigmented epithelium (RPE) expresses clu as well, it is possible that this expression occurred through RPE contamination of the rod single-cell preparation (Trimarchi et al., 2007). A summary of these data can be found in Table 1. Verification of the expression pattern of these genes by another method is required, such as in situ hybridization on retinal sections, as was done for a subset in this study (below).

**Table 1:** Summary of Müller Glia Specific and Enriched Genes^1^

Category	Gene name	Reference	Verification; Specificity	Expression level
Classical markers	ApoE	Amaratunga et al., 1996	ISH	High
	Aqp4	Nagelhus et al., 1998	ISH, DISH	High
	Clu	Blackshaw et al., 2004	ISH, DISH	High
	Vimentin	Greenberg et al., 2007	ISH	High
	Kir2.1	Newman and Reichenbach, 1996	N/A	High
	Kir4.1	Newman and Reichenbach, 1996	N/A	High
	S100a16	Seigel et al., 1996	N/A	High
	Glul	Blackshaw et al., 2004	ISH	High
	CRLBP-1	Matsuda et al., 2004	ISH, DISH	High
	Dkk3	Blackshaw et al., 2004	ISH, DISH	High
	Chx-10	Rowan et al., 2004	N/A	High
New markers	Spbc25		ISH	High
	Itm2b		ISH, DISH	High
	Apg4b		ISH, DISH	Low
	Dbi		ISH	High
	GPR37	Blackshaw et al., 2004	ISH, Xgal	High
	Car2		ISH	High
Müller glia enriched	Abca8a		N/A; high	High
	Sox2	Blackshaw et al., 2004	N/A; high, also prog	High
	Gnai2		N/A; high	High
	Cav		N/A; high	High
	Jagn1		N/A; high	High
	2310032F03Rik		ISH; high	High
	Synpr		N/A; medium	High
	Col9a1		N/A; medium	High
	Prss2		N/A; high, subset	High
	Ube1c		N/A; high, subset	High
	Gnb1l		N/A; high, subset	High
	Bing4		N/A; high, subset	High
	Kcnj10		N/A; high	High
	Cts0		N/A; high	High
	Ctsh		N/A; high	High
	Rhpn		N/A; high	High

1 Literature references for known Müller glia markers are indicated.

Abbreviations: DISH, dissociated cell in situ hybridization; ISH, in situ hybridization; N/A, not done; prog, progenitor cells.

### Identification of genes characteristic for Müller glial cells

To identify Müller glial enriched or specific genes, the analytical tool Cluster 3.0 (Eisen et al., 1998) and Treeview software (Eisen et al., 1998) were used for clustering the single cell profiles, as well as to create a visual map of the data. The five Müller glial cells were once again compared with immature amacrine and ganglion cells and with rod photoreceptors. However, instead of considering only the most highly expressed transcripts, as was done in the previous analysis (Fig. 2), the complete data set obtained from the microarrays was used for comparison. The data were filtered such that any probe set that failed to reach a signal of 1,000 in any single cell was eliminated. The data for the remaining probe sets was log transformed and normalized according to Eisen et al. (1998).

**Fig. 3:**
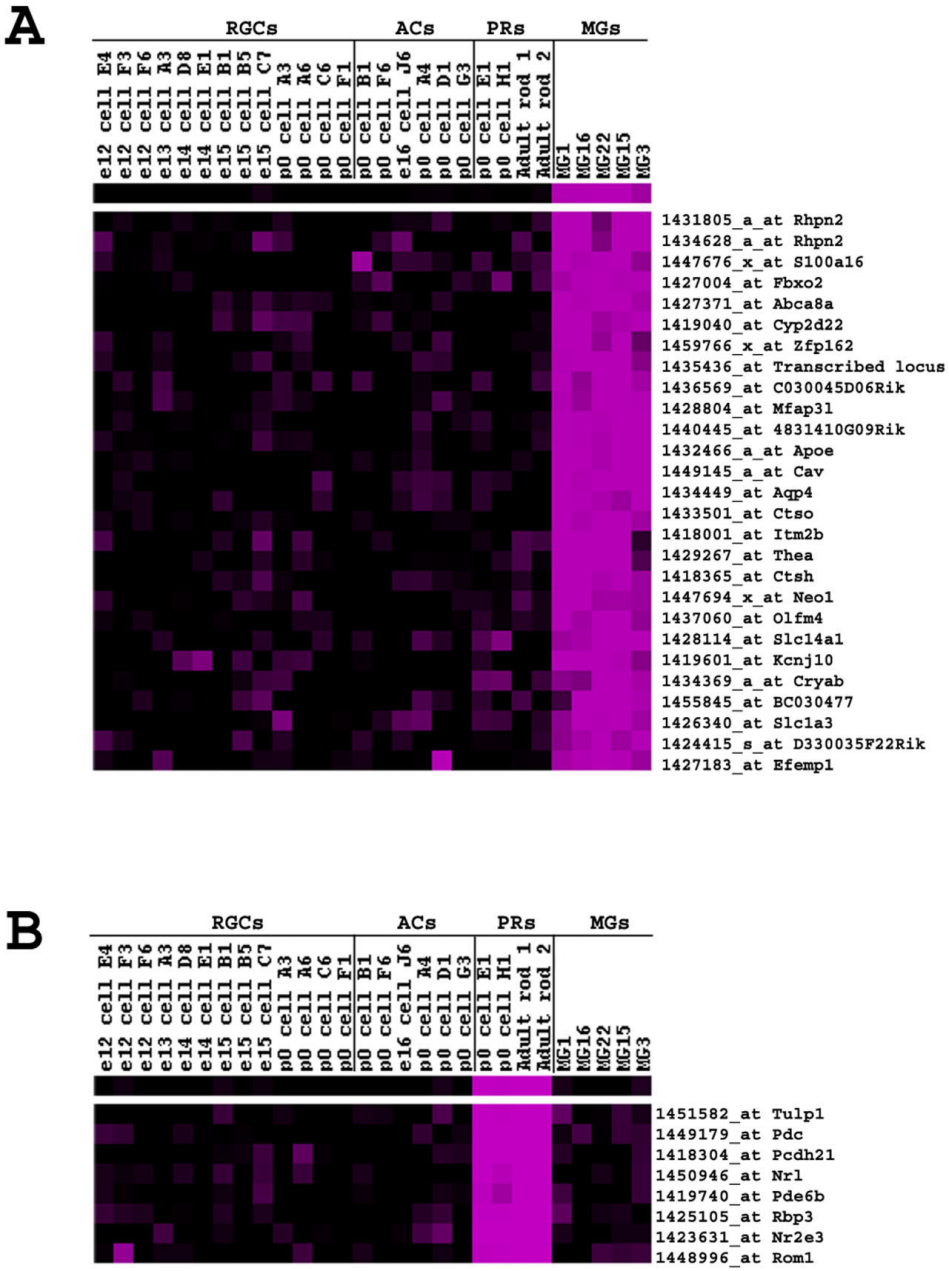
Cluster analysis of retinal single cells. A hierarchical cluster analysis was run on the Affymetrix signals from 19 developing amacrine (ACs) and ganglion cells (RGCs), 2 immature rod photoreceptors (PRs) from postnatal day 0 (P0), 2 mature rod photoreceptors, and 5 mature Müller glial cells (MGs). Selected portions of clusters are shown as a heatmap. Columns represent the samples, and rows represent the transcripts (with abbreviated name and Affymetrix ID). **A**: Part of the cluster around the gene Aqp4, revealing genes enriched in Müller glia. **B**: A cluster of photoreceptor specific genes.

A representative part of the resulting expression level matrix is shown in Supplemental Figure S1 and a portion of that same cluster around Aqp4 is shown enlarged in Figure 3A. Interestingly, tight clustering of genes in a given cell type can be observed, with Figure 3A showing a portion of a Müller glial cluster, and Figure 3B showing a rod photoreceptor cluster. All Müller glial cells shared a similar gene expression profile over a wide range of genes (Supplemental Fig. S1). Other cell types showed expression values that were considerably distinct from those of the Müller glia. The data in Figure 3A of genes enriched in Müller glia suggest a Müller glia core transcriptome, which likely comprises genes encoding some of the key functions of Müller glia. One of the most prominent examples is the inwardly rectifying potassium channel (Kcnj10). Müller glia have been previously shown to play an active role in regulating retinal functions by allowing transport of small molecules through specific ion channels. Other potassium channels expressed in Müller glial cells include Kir4.1 and Kir2.1 (Newman and Reichenbach, 1996; Bringmann et al., 2000). Genes included in this cluster are also ApoE and Abca8a, which have been identified to be among the most highly expressed genes in the previous analysis (Fig. 2). Genes not previously identified in Müller glia, such as two members of the lysosomal cystein protease family, cathepsin O (Ctso) and cathepsin H (Ctsh), and rhophilin, which is involved in actin cytoskeleton reorganization, have been found to be present in this cluster as well.

The expression of genes known to encode functions that might be relevant for Müller glia physiology was examined. For example, as noted above, there are retinal progenitor genes expressed in Müller glia, such as Dkk3 and Chx10 (Blackshaw et al, 2004; Rowan and Cepko, 2004), perhaps in keeping with their ability to produce neurons in some species under some conditions, or respond to injury by re-entering the cell cycle.

Expression of the paired-type homeobox, Pax6, was noted in four out of five of the single Müller glial cells (Fig. 5). Pax6 is a marker for progenitor cells and also for ganglion cells, amacrine cells, and horizontal cells in the mature retina. It has not been reported to be expressed in mature Müller glia. However, expression is consistent with the expression of other progenitor genes in Müller glia.

**Fig. 4:**
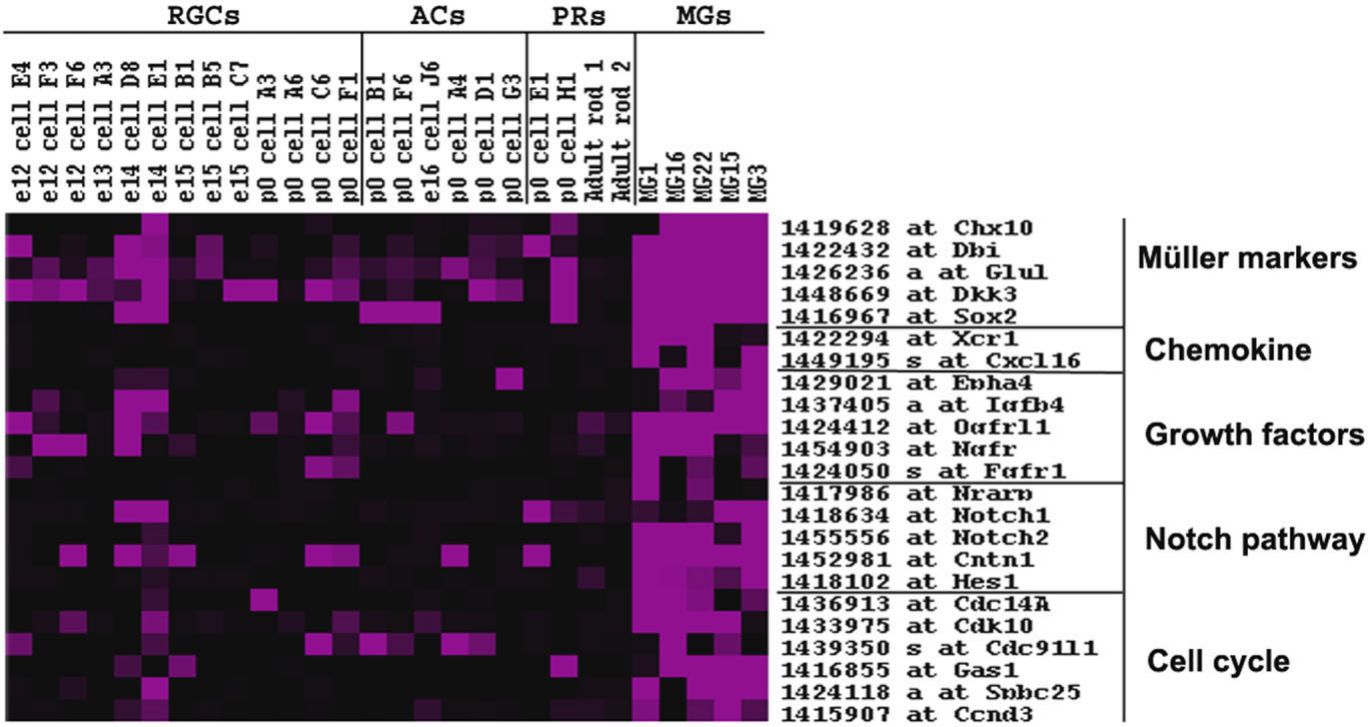
Heatmap of Müller glia enriched genes illustrating potential functions of Müller glia. Expression of genes with potentially relevant functions (such as growth factors, cell cycle components, and Notch pathway components) for Müller glia physiology was examined. The data are summarized as a heatmap. RGCs, retinal ganglion cells; ACs, amacrine cells; PRs, photoreceptors; MGs, Müller glia cells.

Examination of the expression of cell cycle genes by Müller glia showed that cyclinD3, Cdc14A, Cdk10, and Spbc25 were enriched in Müller glia. Several genes of the Notch pathway were expressed in significant amounts in Müller glia, such as Notch1 and Notch2, and might regulate the re-entry of Müller glia into the cell cycle under specific (e.g., pathological) conditions (Fig. 4). Growth factors such as Fgfs have been suggested to be important for short-range interactions among retinal cells. The facts that aFGF can be released from rod outer segments by a phosphorylation-dependent mechanism, and that apical processes of Müller glial cells surround the photoreceptors, suggest that these factors may mediate communication between glial cells and neurons (Mascarelli et al., 1991). Fgfs, neurotrophic factors, and growth factor receptors are also suggested to be important in Müller glial proliferation (Milenkovic et al., 2003). Activation of several growth factor- and G-protein-coupled receptors has been shown to induce proliferation in cultured Müller glial cells (Ikeda and Puro, 1994). FGF r1, Ngf R, and Ogfr were highly expressed among Müller glia, whereas Eph and Igfb4 were expressed among a few Müller glial cells.

**Fig. 5:**
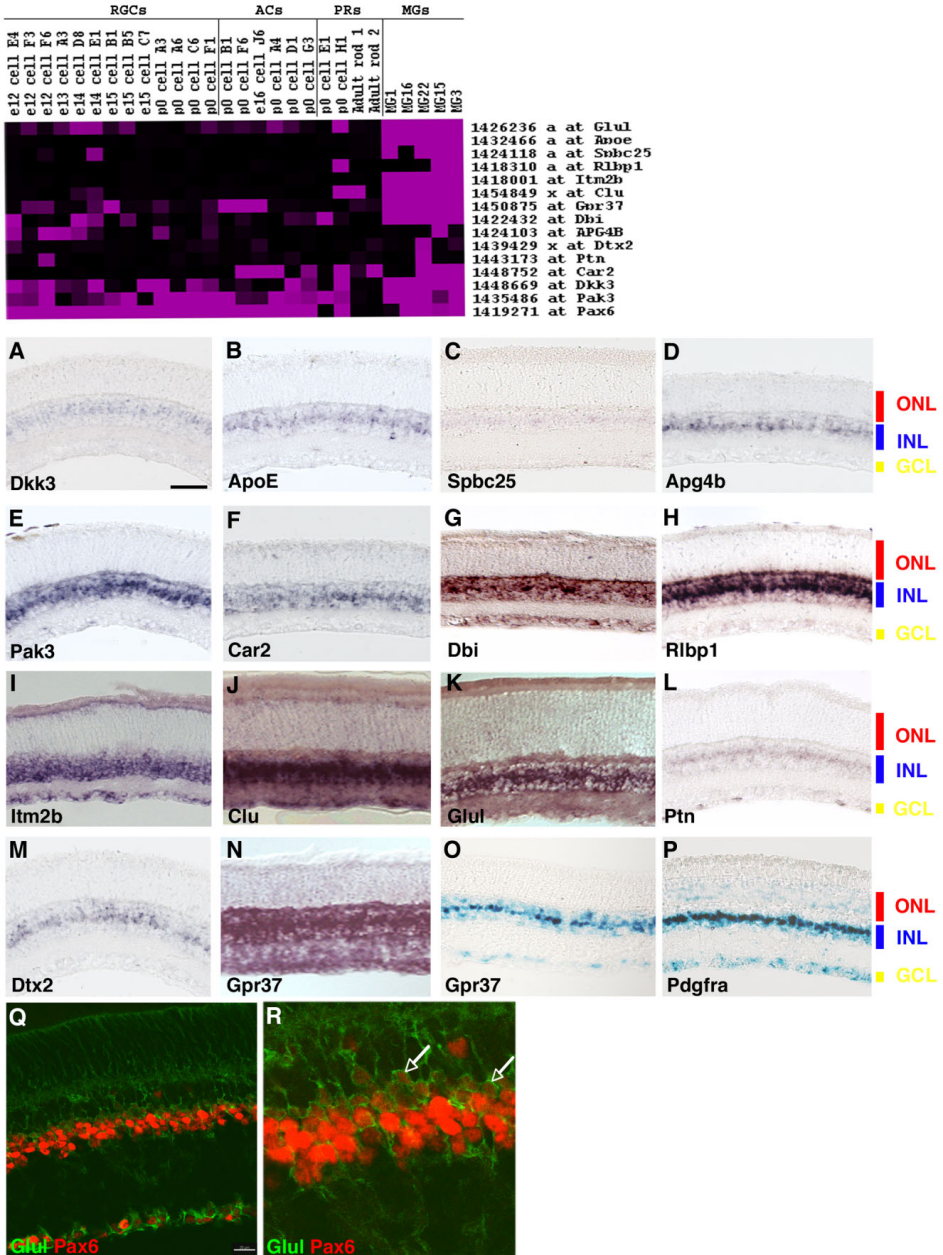
Section in situ hybridization of genes enriched in single Müller glia. **Top**: Heatmap of Müller glia enriched genes. **A–N**: ISH signal of candidate Müller glial transcripts on mouse retinal sections. Gene name is as indicated. Probes used are listed in the supplementary data. **O**: Xgal histochemistry on GPR37^tm1Dgen^ retinal sections. **P**: Recombinase-dependent nuclear lacZ expression in Müller glial cells of *Pdgfra-Cre; RC::PFwe* retinal sections. **Q**: Immunohistochemistry on mouse retinal sections with anti-Pax6 (red) and anti-Glul (green) antibodies. **R**: Digital magnification of image shown in Q. RGCs, retinal ganglion cells; ACs, amacrine cells; PRs, photoreceptors; MGs, Müller glia cells; ONL, outer nuclear layer; INL, inner nuclear layer; GCL, ganglion cell layer. See Supplemental Figure S3 for magenta-green versions of Q and R. Scale bar = 50 μm in A (applies to A–P); 20 μm in Q.

Under pathological conditions, Müller glia might additionally act as modulators of the immune and/or inflammatory response. Retinal glial cells have been shown to be a major source of cytokines after retinal detachment (Nakazawa et al., 2006). Recently, MCP-1 (monocyte chemoattractant protein 1) expression was shown in Müller glia after retinal detachment (Nakazawa et al., 2007). Components of the chemokine system that could be detected in Müller glia were Xcr1 and Cxcl16 (Fig. 4). Müller glia might also be involved in the prompt clearing of photoreceptor debris under pathological conditions. During *Drosophila* development, it has been shown that glial cells engulf degenerating axons through recognition mediated by drpr/ced-1 and ced-6/CED-6 (Awasaki et al., 2006). It has been proposed that apoptotic cells and degenerating axons of mature neurons are removed by a similar mechanism (Awasaki et al., 2006; MacDonald et al., 2006). The ability of Müller glia to phagocytose retinal cell debris and foreign substances has been previously shown in vitro (latex beads) and in vivo (melanin) after retinal detachment (Mano and Puro, 1990; Stolzenburg et al., 1992; Francke et al., 2001). However, two of the genes, Megf10 (multiple EGF-like-domains 10)/ced-1 and Abca1 (ATP-binding cassette, subfamily A, member 1)/ced-7 were observed to be expressed in a subset of Müller glia, whereas others, Gulp1 (GULP: engulfment adaptor PTB domain containing 1)/ced-6, Lrp1 (low-density lipoprotein receptor-related protein 1)/ced-1, and Rac1 (RAS-related C3 botulinum substrate 1)/ced-10, could not be detected in the Müller glial cells studied here.

### Heterogeneity of Müller glia

Although the focus of this study was to define the transcriptome of Müller glia, it was noted that certain genes were expressed heterogeneously among the Müller glial cells analyzed (Fig. 4, Supplemental Figs. S1, S2), including common housekeeping genes such as cytoplasmic β-actin, β-2-microglobulin, heat shock protein 1 β, and TATA box binding protein. This might be due to technical or real differences. Indeed, other studies employing Affymetrix microarrays to examine housekeeping gene expression among different tissues, as well as our SAGE study of the retina during development, similarly found that housekeeping genes vary considerably in their expression levels (Warrington et al., 2000; Blackshaw et al., 2004; Lee et al., 2007).

Little is known about the significance and extent of Müller glial cell heterogeneity, although it is clear that molecular differences exist because the homeodomain-containing transcription factor Chx10 is expressed in only a subset of all Müller glial cells (Rowan and Cepko, 2004). Heterogeneous expression of Chx10 could also be observed in the current study (Fig. 4). Heterogeneity of gene expression among three of the Müller glial cells was observed across a large cluster (Supplemental Fig. S1). This was further analyzed for a few of these genes by using in situ hybridization (see below). A recent expression analysis of astrocytes has revealed extensive molecular heterogeneity among that class of glial cells as well (Bachoo et al., 2004). Furthermore, morphological studies have identified extensive anatomical differences between Müller glial cells in the central versus peripheral chick retina (Anezary et al., 2001). Further characterization of the glial genes identified in this study will help clarify the diversity of Müller glial cells and perhaps shed light upon a potential functional diversity.

### Müller glia enriched genes: Fisher's exact test

To examine the relationships of gene expression patterns by using a method other than hierarchical clustering, the data were subjected to an analysis using the Fisher's exact test (Trimarchi et al., 2007). This test calculates the probability that a particular expression pattern for a pair of genes across a number of arrays would occur by chance. Supplementary Table T3A shows the genes that have similar a pattern to that of ApoE, with *P* values more significant than 10^−4^. This method identified many of the same genes identified by using hierarchical clustering around ApoE. The Fisher's exact test was also applied to look for genes with a similar distribution to that of Aqp4 (Supplementary Table T3B). As expected, very similar genes were obtained as for ApoE, confirming the observation that ApoE and Aqp4 are enriched/specific in Müller glia. Some highly expressed and potentially Müller glia specific genes shown and discussed in Figure 2 could be identified through this method as well.

### Validation of expression in Müller glia

To validate the expression of genes expressed in Müller glial cells, in situ hybridization on retinal sections was performed for a subset of genes (Fig. 5). The corresponding Affymetrix signal values are represented in the top panel of Figure 5 as a heatmap. Because the cells used for comparison were mainly immature cells, some of the genes that are indeed specific to Müller glia in the adult retina are also expressed in those immature cells that still contain a certain expression profile of (late) progenitor cells. The similarity of Müller glia gene expression to progenitor cell gene expression has been discussed previously (Fig. 4 and Blackshaw et al., 2004). Müller glia transcripts were localized most prominently in the middle portion of the inner nuclear layer (INL), consistent with the location of the transcript in the cell body. Transcripts with this predicted restriction in expression pattern are Dkk3, ApoE, Spbc25, Apg4B, Pak3, Car2, Rlbp1, deltex 2 homolog (Dtx2), and pleiotrophin (Ptn) (Fig. 5A–F,H,L,M). Certain genes, such as Dbi, GPR37, and Glul (Fig. 5G,N,K, respectively), gave punctuate staining in the middle portion of the INL, as well as diffuse expression in the outer nuclear layer (ONL), consistent with expression in ascending glial processes, and in the ganglion cell layer (GCL), consistent with expression in Müller glial descending processes and endfeet. Expression in the INL was either throughout the INL, as is the case for clu (Fig. 5J), or in a subset of cells in the middle of the INL, as is the case for Dbi (Fig. 5G). The pan INL pattern is suggestive of a gene being expressed in several cell types in the INL, whereas the more specific middle of the INL pattern is more consistent with expression restricted to Müller glia. Even transcripts expressed in only a subset of Müller glial cells profiled, such as Apg4b, Dtx2, and Ptn could be verified and detected by in situ hybridization (Fig. 5D,M,L, respectively).

**Fig. 6:**
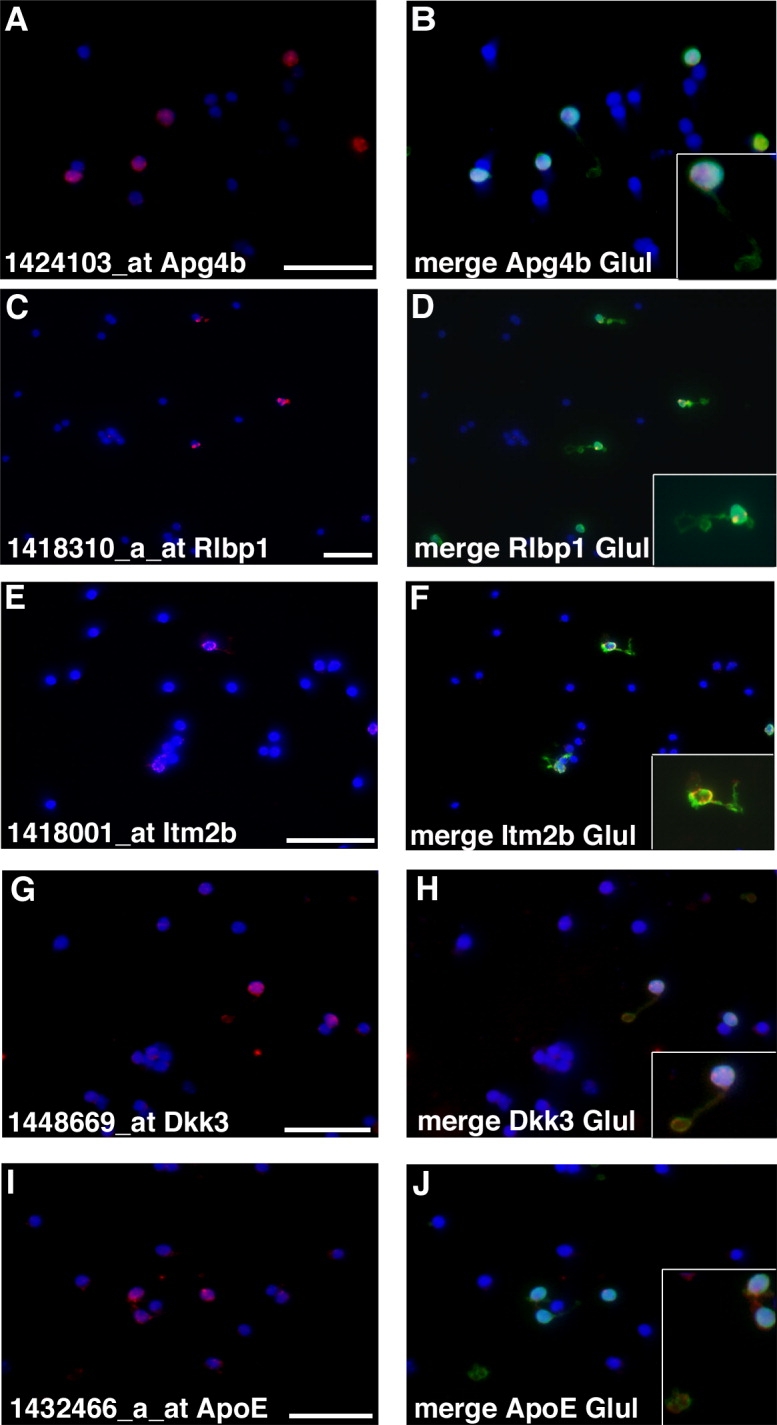
Dissociated cell ISH confirms expression in Müller glia. **A–J**: Retinae from mature mice were dissociated, plated on slides, and processed for detection by ISH for the indicated probes (ApoE, Dkk3, Apg4b, Itm2b, Rlbp1). Merged images show DAPI stain and immuno-staining for Glul and ISH. Insets are digital magnifications. Scale bar = 25 μm in A (applies to A,B), C (applies to C,D), E (applies to E,F), G (applies to G,H), and I (applies to I,J).

It was of interest to determine whether some of the genes with moderate to high levels of Affymetrix signals in profiled Müller glia were specific to Müller glia. Specificity might give some insight into function and would indicate that such genes are suitable for use as specific markers of Müller glia. Although analysis of candidate Müller glial genes by section ISH is a fairly rapid and informative assay, it was unclear for some genes whether their expression was restricted to Müller glial cells. To determine specificity of expression more precisely, ISH on dissociated cells was carried out in combination with immunocytochemistry for the known glial marker glutamine synthetase (Glul). Dissociated cell in situ hybridization (DISH) for Itm2b revealed that it was exclusively expressed in Glul-positive cells. A modulator of the Notch pathway, Apg4b, as well as Dkk3, Aqp4, and ApoE were shown to be fairly specific to Müller glia as well (Fig. 6). DISH also allowed an assessment of heterogeneity of expression within Muller glial cells. The Rlbp1 transcript, expressed in only three of five cells in the microarray analysis, was found in only a subset of Müller glia, in 70% of the cells marked with the anti-Glul (Supplemental Fig. S2). In contrast, there was a complete overlap of Itm2b and clusterin transcript expression with anti-Glul staining (Fig. 5, top panel, Supplemental Fig. S2).

Additional validation of GPR37 as a Müller glia specific gene in the murine retina was possible through the use of a transgenic mouse line, GPR37^tm1Dgen^, available from the Jackson Laboratory. The GPR37^tm1Dgen^ mice express a transgene consisting of lacZ driven by the endogenous GPR37 promotor. Xgal staining on retinal sections showed lacZ expression in Müller glial cells (Fig. 5O).

Pax6 expression was further investigated by immunocytochemistry by using anti-Pax6 and anti-Glul antibodies (Fig. 5Q,R; see Supplemental Fig. S3 for magenta-green versions of images). Analysis of the sections with confocal microscopy showed that Pax6 is indeed expressed in adult Müller glial cells, as well as in some types of retinal neurons, as previously reported for neurons (de Melo et al., 2003), but not previously reported for Müller glial cells.

### Creation of a mouse line expressing Cre for genetic access to Müller glia

To create a line of mice for future experiments in which gene expression specifically in Müller glial cells could be manipulated, a BAC was engineered to express Cre from a promoter that was predicted to be enriched or specific to glial cells (Kessaris et al., 2006). This BAC was used to create a transgenic mouse. Mice that successfully transmitted the BAC were assessed for Cre activity by crossing to mice carrying a nuclear lacZ floxed indicator allele, *RC::PFwe* (Farago et al., 2006). Strong nuclear lacZ expression was detected in all or nearly all Müller glial cells in the progeny of this cross (Fig. 5P). This may be a fortuitous finding, as inspection of the microarray data for Müller glia did not show expression of Pdgfra. Some Xgal staining was observed in the ONL and GCL, but it was not distinctly nuclear. Control Xgal staining of littermates did not show this lacZ activity, and thus the origin of the ONL and GCL staining is unclear.

## DISCUSSION

The goal of this study was to define the transcriptome of mature Müller glial cells. A single cell profiling approach was used because total tissue preparations of the retina would only contain approximately 5% of cells as Müller glia (based on the percentage of cells showing Glul immunoreactivity after dissociation into single cells). A PCR-based amplification method was used to generate enough cDNA from a single cell to use as probe on Affymetrix microarrays. The microarray results were then validated by section ISH and DISH. Validation was particularly important because PCR-based protocols may lead to a higher false-positive rate, compared with the higher false-negative rate that is generally associated with linear amplification methods (Van Gelder et al., 1990; Iscove et al., 2002; Che and Ginsberg, 2004; Subkhankulova and Livesey, 2006). The microarray data from the single cells confirmed the high expression of many known Müller glial genes and also revealed the expression of many genes not previously known to be transcribed in Müller glia. By characterizing single, freshly isolated Müller glial cells, this study also circumvented the need to use FACS or in vitro culture, or to manipulate these cells further, and thereby minimized induced gene expression changes. We have found this method to be fairly sensitive and reproducible in the current study, as well as a previous study of developing amacrine and ganglion cells (Trimarchi et al., 2007).

We also created and characterized a *Cre* line for recombination and expression in Müller glial cells. This novel line will provide genetic access for manipulations of Müller glia in mice, in addition to the recently published lentiviral vector-based approach applied in rats by Greenberg et al. (2007). The utility of such methods might be especially interesting in retinal disease backgrounds.

### Relationship of Müller glia to progenitor cells and stem cells

A SAGE study, coupled with large-scale in situ hybridizations, showed that genes enriched or specific to Müller glia in the mature retina are also expressed in multipotent retinal progenitor cells (Blackshaw et al., 2004). Similarly, a microarray analysis of genes enriched in 4N retinal progenitor cells showed enrichment for genes expressed in Müller glia, relative to retinal neurons (Livesey et al., 2004). In addition, some genes expressed at a relatively high level in mature Müller glia, such as Glul and Car2, are also expressed in late retinal progenitor cells. To this list of progenitor genes expressed in Müller glia, we can now add Pax6, a gene required for formation of the retina and other ocular structures (Hill et al., 1991; Glaser et al., 1992). This extensive overlap in gene expression between Müller glial cells and progenitors, coupled with the observation that Müller glia can divide in response to some stimuli, might indicate that Müller glia are a form of retinal progenitor cell.

Late retinal progenitor cells might become Müller glia without an irreversible cell fate determination event, such as occurs when a neuron is generated. Progenitor cells becoming Müller glia might retain much of the progenitor gene expression program, including those genes that allow a cell to divide (Fig. 3C), whereas upregulating and/or initiating expression of genes required for specialized Müller glial functions, such as ApoE, Glul, and GFAP. Candidate genes for regulation of this transition from retinal progenitor cell to Müller glia are the genes Notch1, Hes1, Hes5, and Rax, as overexpression of each of these genes in retinal progenitor cells leads to production of cells with at least some properties of Müller glia (Bao and Cepko, 1997; Furukawa et al., 2000; Hojo et al., 2000; Jadhav et al., 2006a,b). In contrast, overexpression of positive bHLH genes, such as Math 3, NeuroD, and many others, leads to a reduction in Müller glial production (Morrow et al., 1999; Cai et al., 2000; Inoue et al., 2002). The transition of a late progenitor cell to a Müller glia cell might thus depend on the balance of positive and negative bHLH genes, which might normally be regulated by Notch signaling. It appears that Notch activation generally favors formation of cells with glial characteristics not only in the retina, but also elsewhere in the nervous system (Morrison et al., 2000; Gaiano and Fishell, 2002). The potential of Müller glial cells to produce neurons in the adult retina might suggest that a discrete set of genes limits this ability.

In light of this notion, it is interesting to consider the Notch pathway genes further, not only as regulators of the formation of Müller glia as discussed above, but also as regulators of the re-entry into the cell cycle and/or as negative regulators of the production of neurons by Müller glia. The single-cell profiles for mature Müller glia include Notch1, Notch2, Hes1, Nrarp, and Cntn1, as well as regulators of Notch signaling, such as (Fig. 4), which bear examination in this context. Hes1, a downstream target of NICD, is also highly expressed by neural stem cells in the forebrain (Ohtsuka et al., 2001; Chojnacki et al., 2003), perhaps reflecting a similar role.

### Similarity to other glial cell types

A comparison of the Müller glia transcriptome with those of other glial cell types of the central nervous system (Bachoo et al., 2004) failed to reveal common clusters of gene expression. This might be due to technical differences or might suggest a different genetic constitution among them. However, there were some transcripts with shared expression among Müller glia and other glial cell types. Müller glia enriched transcripts that are also enriched in astrocytes are peroxiredoxin 6, Basigin (also called CD147), caveolin, CD9 antigen, carboxypeptidase E, cystatin C, phospho protein enriched in astrocytes (Pea15), Ptn, Ret4, Slc2a1, and Slc3a2 (Bachoo et al., 2004; Vincent et al., 2005). Preliminary comparisons also imply that Müller glial cells are less related to Schwann cells (peripheral glia), because many transcripts highly expressed in Müller glia are depleted in Schwann cells, i.e., ApoE, clu, cystatin C (Cst3), enolase1 α (Eno1), Gapd, Glul, malate dehydrogenase (Mdhl), and myotrophin (Mtpn) (Vincent et al., 2005).

Lastly, on the basis of the investigation presented here, we hope to continue our efforts to understand better the role(s) of Müller glia in retinal degeneration. Several diseases of the retina, such as retinitis pigmentosa and macular degeneration, lead to a severe loss of photoreceptor neurons and involve “activation” of Müller glia. The identification of the cellular events and changes that take place within Müller glia during the progressive neuronal cell death might be applicable to a therapeutic strategy.
